# Development of Multiplex PCR assay for detection of Canine Infectious Respiratory Disease Complex (CIRDC) pathogens in dogs

**DOI:** 10.3389/fcimb.2025.1661499

**Published:** 2025-11-28

**Authors:** Ritik Kaul, Jai Bhagwan, Kanisht Batra, Parveen Kumar, Divya Agnihotri

**Affiliations:** 1 Department of Veterinary Medicine, Lala Lajpat Rai University of Veterinary and Animal Sciences, Hisar, Haryana, India; 2 Department of Animal Biotechnology, Lala Lajpat Rai University of Veterinary and Animal Sciences, Hisar, Haryana, India; 3 Department of Veterinary Microbiology, Lala Lajpat Rai University of Veterinary and Animal Sciences, Hisar, Haryana, India

**Keywords:** canine infectious respiratory disease complex (CIRDC), multiplex PCR, sensitivity, specificity, dogs

## Abstract

**Background:**

Canine Infectious Respiratory Disease Complex (CIRDC) is a highly contagious, multifactorial syndrome that primarily affects dogs in crowded environments such as shelters, kennels, and breeding facilities. Three major CIRDC-associated pathogens: Canine distemper virus (CDV), Canine adenovirus type 2 (CAV-2), and *Bordetella bronchiseptica* (Bb) have been reported in the canine population of India.

**Materials and Methods:**

In this study, a multiplex PCR (mPCR) assay was developed and optimized for the simultaneous detection of these three pathogens. The multiplex assay was designed targeting three genes H, E3 and *bfrZ* of CDV, CAV-2 and Bb respectively. This multiplex assay was optimized in both singleplex and multiplex formats by adjusting key PCR parameters such as primer concentration, annealing temperature, and incubation time to achieve distinct and reproducible amplification of all three targets.

**Results:**

The developed assay demonstrated high analytical sensitivity, detecting 1,060 copies/μL for CDV, 11,403 copies/μL for CAV-2, and 11,016 copies/μL for Bb, with 100% specificity and no cross-reactivity with non-target organisms. The assay was validated on 55 clinical samples of dogs suspected with CIRDC, the assay detected pathogens in 32.2% of cases, with CDV being the most prevalent (25%). Compared with previously published singleplex PCR methods, the mPCR showed excellent diagnostic performance, achieving 94.12% sensitivity, 94.74% specificity, and 94.55% overall accuracy.

**Conclusion:**

This study demonstrates a rapid, specific, and cost-effective diagnostic mPCR assay capable of efficiently identifying key CIRDC pathogens in a single reaction. The assay is highly suitable for molecular diagnosis as well as large-scale field surveillance.

## 1 Introduction

Canine Infectious Respiratory Disease Complex (CIRDC) or Kennel cough or Infectious tracheobronchitis, is a multifactorial respiratory disease in dogs, occurring globally and is marked by its high contagiousness and swift transmission (Yondo et al., 2023). It affects the upper and lower respiratory tract including nasal mucosa, trachea, larynx, bronchi (Buonavoglia and Martella, 2007). A notable characteristic of CIRDC is its propensity to result in collective infections and is prominent in environments where dogs are kept in groups or interact with other dogs, particularly in kennels, dog training facilities and animal shelters (Zhao et al., 2024). Common clinical signs include a harsh cough, serous ocular or nasal discharge, and sneezing, with normal energy and appetite. Severe signs like fever, lethargy, or appetite loss suggest secondary bacterial infections (Reagan and Sykes, 2020). Traditionally, the main pathogens linked to CIRDC include *Bordetella bronchiseptica*, canine adenovirus type-2, canine distemper virus, canine herpes virus and canine parainfluenza virus (Day et al., 2020; Wille et al., 2020). The primary causal agent of CIRDC such as *B. bronchiseptica (*Bb*)*, Canine adeno virus type 2 (CAV-2) and Canine distemper virus (CDV) have been reported in Indian dog population and are frequently associated with severe respiratory distress (Keil and Fenwick, 1998; Erles et al., 2004; Posuwan et al., 2010; Radtanakatikanon et al., 2013; Sariga et al., 2022).

*Canine distemper virus*, a *Morbillivirus* in the *Paramyxoviridae* family, is a highly contagious pathogen affecting the respiratory, gastrointestinal, and nervous systems. Spread via aerosolized secretions, CDV initially targets lymphoid tissues before systemic dissemination (Reagan and Sykes, 2020). Clinical signs in this disease may vary from subclinical in initial stages to severe, including cough, ocular discharge, gastrointestinal upset and neurologic symptoms. Shedding begins around day five post-infection and may continue for up to four months (Martella et al., 2008).

*Canine adenovirus type 2*, a non-enveloped, double-stranded DNA virus of the *Adenoviridae* family, causes mild respiratory disease in dogs. It infects epithelial cells of the nasal mucosa, pharynx, bronchioles, and alveoli, with clinical signs including sneezing, nasal discharge, and a dry cough. Severity increases in co-infections with other CIRDC pathogens. Shedding typically lasts 1–2 weeks, but the virus may persist environmentally for weeks to months (Reagan and Sykes, 2020).

*Bordetella bronchiseptica* is a Gram-negative coccobacillus that colonizes the upper respiratory tract and causes respiratory disease in dogs. This bacteria is zoonotic in nature and has been found in other species also including cats, pigs, rabbits, and humans (Islahi et al., 2019). It is highly contagious, transmitted via aerosol droplets, with an incubation period of 2–10 days (Mattoo and Cherry, 2005). Clinical signs may range from mild nasal discharge, sneezing, and a dry, honking cough to severe lower respiratory illness with lethargy, fever, and a productive cough (Hozbor et al., 1999). Shedding can persist for over a month, and in some cases, several months (Ellis, 2015).

Diagnosing CIRDC-associated pathogens is essential for determining appropriate treatment, prognosis, and prevention strategies. The traditional approach to diagnosing canine infectious tracheobronchitis is isolating the causative organism from nasal and throat swabs of infected dogs using cultural and biological techniques, followed by identification through biochemical, serological and molecular methods (Hozbor et al., 1999). Due to the time-consuming nature, limited specificity or sensitivity of many diagnostic tests and the involvement of multiple pathogens, an approach of multiplex PCR is a suitable method for detecting CIRDC pathogens (Payungporn et al., 2006; Suwannakarn et al., 2008; Jeoung et al., 2013; Pecoraro et al., 2013). This multiplex method allows simultaneous detection of multiple pathogens in a single tube, which is particularly valuable for cases involving co infections. Multiplex PCR offers high sensitivity and specificity, enabling the detection of both bacterial and viral pathogens in a single PCR reaction with high accuracy than conventional methods of detection. It is also very useful in developing countries with few resources for diagnosis (Piewbang et al., 2016). Therefore, a novel Multiplex PCR (mPCR) assay was developed for the concurrent detection of Canine distemper virus (CDV), Canine adenovirus type 2 (CAV-2) and *Bordetella bronchiseptica*.

## 2 Materials and methods

### 2.1 Gene selection and primer designing

For the development of mPCR assay targeting Hemagglutinin gene (H gene) of CDV, Early region gene (E3 gene) of CAV-2 and Bordetella filamentous hemagglutinin related gene Z (bfrZ gene) of *Bordetella bronchiseptica* were selected, respectively. Primers were designed using ClustalW and Mega 10.2 version softwares, with conserved regions identified via sequence alignment of reported GenBank accessions no. such as KC479140, KC479141, LC011103, KC479138, MF964181 (CDV), OP618116, OP644981, MT892837, S38212, U77082 (CAV-2) and AJ251793, CP132332, BX640451, LR134326 (Bb) (**Supplementary Table S1**). Primer properties were checked *in silico* using NCBI BLAST and commercially synthesized by IDT, India (**Table 1**).

**Table 1:** Details of self-designed primers for mPCR assay.

Primer name and position (nucleotide)	Primer sequence (5’-3’)	Primer size (bp)	Expected product size
CD/H/F/346-368	GTGACATATTCCCACCATACAG	22	274 bp
CD/H/R/597-619	AGTTGGTTGTCTGGAGTAATGG	22	
CAV2/E3/F/38-58	CTCTTCCCAGCGTAACCATA	20	451 bp
CAV2/E3/R/464-488	TGGCTCTGCAAGTTACTCTAAATA	24	
BBV/BfrZ/F/1519-1538	GTTCAGGTCATTGCGTTTG	19	672 bp
BBV/BfrZ/R/2170-2190	GACGACCAGGATCACATCTT	20	

**Table 3:** Sex- wise occurrence of CIRDC pathogens in dogs.

Sex	Total number of CIRDC cases (n=55)	% occurrence
Male	38	69.09
Female	17	30.90

**Table 4:** Age-wise occurrence of CIRDC pathogens in dogs.

Age group	Total number of CIRDC cases (n=55)	% occurrence
0–6 months	10	18.18
6–12 months	08	14.54
1-3years	26	47.27
3-6year	04	7.27
>6years	07	12.72

**Table 5:** Occurrence of CIRDC in dogs based on vaccination status.

Vaccination status	Total number of CIRDC cases (n=55)	% occurrence
No vaccination	12	21.81
Incomplete vaccination	24	43.63
Complete vaccination	19	34.54

### 2.2 Extraction from vaccine for generation of positive controls

CDV and CAV-2 were obtained from live attenuated freeze-dried vaccine Canishot^®^ DHPPL (Intas Pharmaceuticals, India) and Bb from live freeze-dried vaccine Nobivac^®^ KC (MSD Animal Health, India).

### 2.3 Nucleic acid extraction, quantification and reverse transcription

The extraction of DNA from vaccine was using the DNA extraction kit (HiPurA^®^ mammalian Genomic DNA Purification Kit, Kennett Square, PA, USA) according to manufacturer’s recommendation and TRIzol method was used for RNA extraction. Briefly, DNA extraction was done by adding 200 μL of vaccine, 200 μL HiPurA^®^ Genomic lysis/binding buffer, 20 μL of Proteinase K. This mixture was incubated at 55 °C for 1 hour in water bath. After incubation, 200 μL of 100% ethanol was added and homogenous mixture was carefully poured in the HiPurA^®^ spin column in a 2 ml collection tube and centrifuged at 12000 rpm for 1 min. The spin column was washed with 500 μL of prewash buffer and 500 μL of wash buffer by successive centrifugation at 12000 rpm for one min. After centrifugation, the DNA was eluted by adding 20 μL of elution buffer in spin column and subsequently extracted DNA was stored at -20 °C for future use. For RNA extraction, 400 μL of vaccine was mixed with 1 mL of TRIzol reagent and vortexed vigorously. In this mixture, 200 μL of chloroform was added followed by vigorous vortexing to avoid formation of insoluble aggregates. This mixture was centrifuged for 15 min at 12,000 rpm at 4°C for phase separation. The aqueous phase was then mixed with equal volume of chilled isopropanol and kept at -20°C overnight. RNA was pelleted by spinning at 12,000 rpm for 20 min at 4°C and the supernatant was discarded. The washing of pellet was done by adding 1 mL of 70% ethanol (chilled) and operated at a speed of 12,000 revolutions per minute continuously at 4°C for 10 minutes. After centrifugation, the pellet was air dried for 2 to 3 hours followed by addition of nuclease free water (NFW). Nucleic acids were quantified using Nanodrop spectrophotometer (Thermo Fisher Scientific) at an absorbance of 260 and 280 nm to derive the A_260_/A_280_ ratio.

The cDNA of the isolated RNA was prepared using iScript cDNA Synthesis kit (BioRad, California, USA) as per the manufacturer’s protocol. Briefly, reaction mixture was made using 4 μL of 5X Buffer, 1 μL of Reverse Transcriptase (RT) enzyme, 5 μL of NFW and 10 μL of extracted RNA (50ng) as template. The PCR conditions for the reaction were 25°C for 5 min, followed by 46°C for 1 hour and inactivation at 95°C for 1 min. The cDNA and DNA were stored at −20 °C until used for further PCR amplification.

### 2.4 PCR amplification and purification of extracted products

The extracted DNA (CAV-2 and Bb) and synthesized cDNA (CDV) were subjected to conventional PCR amplification using self-designed primers specific to each target. The PCR for DNA was performed in Thermal cycler (Sure Cycler 8800, Agilent Technologies) in 12.5 μL reaction containing 3 μL of template DNA, 6.25 μL of 2X GoTaq^®^ Green Master Mix (Promega, Wisconsin, US), 0.4 μM (0.5 μL of 10 μM concentration) each of forward and reverse primers and 2.25 μL of NFW. The PCR for cDNA includes 12.5 μL reaction containing 5 μL of template cDNA, 6.25 μL of 2X GoTaq^®^ Green Master Mix (Promega, Wisconsin, US), 0.4 μM (0.5 μL of 10 μM concentration) each of forward and reverse primers and 0.25 μL of NFW. The cyclic conditions for all the three PCR (CDV, CAV-2 and Bb) were initial denaturation at 95 °C for 2 minutes, followed by 40 cycles of denaturation at 95 °C for 1 minute, annealing at temperatures ranging from 45 °C to 60 °C for 30 seconds (depending on the primer set), and extension at 72 °C for 30 seconds. A final extension step was carried out at 72 °C for 10 minutes to ensure complete amplification of the target sequences. The PCR products generated were then purified using the Monarch^®^ DNA Gel Extraction Kit (New England Biolabs Inc., Massachusetts, US), following the manufacturer’s protocol, to obtain clean gel-extracted amplicons suitable for downstream applications.

### 2.5 Cloning of purified products and plasmid isolation

For generation of positive controls, PCR amplicons of CDV, CAV-2 and Bb were cloned into the pJET1.2/blunt vector (CloneJET™ Kit, Thermo Scientific, Waltham, MA USA) and transformed into *E. coli* DH5α prepared via calcium chloride method. Briefly, a single colony of *E. coli* DH5α was inoculated in 5mL LB broth and incubated overnight at 37°C in shaking incubator at 160 rpm. From the overnight grown culture, culture was inoculated in ratio of 1:100 and incubated at 37°C in shaking incubator until the OD_600_ was between 0.4-0.6. After incubation, the cells were centrifuged at 5000 rpm for 10 min at 4°C for pelleting. This pellet was resuspended in 0.6 volume of ice cold 0.1M MgCl2 (4.8 mL) + 0.1M CaCl2 (1.2 mL) in the ratio of 4:1 followed by incubation on ice for 15 min. Cells were again centrifuged at 5000 rpm for 10 min at 4°C. Supernatant was discarded and cell pellet was again resuspended in 500 μL of ice cold 0.1M CaCl2 and stored at 4°C for further use. The PCR products are treated with blunt-end enzyme and ligated in pJET1.2 vector using T4 DNA ligase. The transformants were selected on LB agar with ampicillin. Recombinant clones were identified by colony touch PCR using gene-specific primers under standardized conditions (95°C for a period of 2 min, denaturation at 95°C for a duration of 1 minute, followed by annealing at 55°C for 30 sec, extension at 72°C for 30 sec and final extension at 72°C for 10 minutes).Plasmids were subsequently isolated using the Monarch^®^ Plasmid Miniprep Kit (NEB, Massachusetts, US) and quantified using Nanodrop spectrophotometer (Thermo Fisher Scientific, Waltham, MA USA). Plasmids were confirmed for presence of targeted genes using automated sanger sequencing (Biokart Pvt LTD).

### 2.6 Optimization of simplex PCR assay

Simplex PCR assays were optimized using the generated plasmid for CDV, CAV-2, and Bb by adjusting annealing temperature, extension/annealing times, and primer concentrations. Reactions were performed in a 12.5 µL reaction volume using 3 µL of simplex plasmid DNA as template, 6.25 µL of 2X GoTaq^®^ Green Master Mix, and 10 µM each of forward and reverse primers, 2.25 µL of NFW. Annealing temperature optimization was conducted using a gradient of 50–60 °C. Time optimization involved three combinations of annealing (15 s, 30 s, 45 s) and extension (15 s, 30 s, 45 s) durations. Primer concentrations were optimized using a 4×4 checkerboard matrix ranging from 0.2 µM-0.8 µM for both forward and reverse primers.

### 2.7 Optimization of multiplex PCR assay

Multiplex PCR assays were developed and optimized for CDV, CAV-2, and Bb by adjusting annealing temperature, extension/annealing times, and primer concentrations. Reactions were performed in a 12.5 µL reaction volume using 3 µL of multiplex plasmid DNA as template, 6.25 µL of 2X GoTaq^®^ Green Master Mix, and 10 µM each of forward and reverse primers, 2.25 µL of NFW for all three targets. Annealing temperature optimization was carried out using a gradient of 45–60 °C. Time optimization involved testing three combinations of annealing (15s, 30s, 45 s) and extension (15s, 30s, 45 s) durations. Primer concentrations were optimized using a checkerboard matrix ranging from 0.1-0.4 µM for both forward and reverse primers.

### 2.8 Analytical sensitivity and specificity, reproducibility

Analytical sensitivity of the multiplex PCR assay was assessed using ten-fold serial dilutions of plasmid DNA, and the lowest dilution consistently yielding a detectable band was used to determine the detection limit. Plasmid copy number was calculated using the formula: Copy number = [AxNo]/ [length (plasmid+ insert size) ×1×10^9^×660], in this formula, A represents the DNA concentration in ng/μl No. is Avogadro’s number (6.022X10^23^) the average weight of a nucleotide base pair (bp) is assumed to be 660 Daltons, and the number of template copies in the sample can be estimated by multiplying the DNA concentration by 1x10^9^ (conversion factor for ng). Analytical specificity was evaluated by testing the assay against target plasmids for CDV, CAV-2, and Bb, as well as the commercial DHPPiL vaccine. Amplification products were analyzed on a 1.5% agarose gel, confirming specific amplification of target sequences without cross-reactivity. Reproducibility was evaluated by measuring both intra-assay and inter-assay variations using positive controls and sequenced clinical samples. Intra-assay variation was assessed by performing triplicate amplifications of templates at 10^−^¹^0^ ng and 10^−^¹ ng per reaction within a single multiplex PCR assay. Inter-assay variation was determined by conducting the same multiplex PCR assay in three independent experimental runs using the same template concentrations.

### 2.9 Diagnostic performance of the multiplex PCR

Oropharyngeal swabs were collected from dogs presented to the Canine Section of the Veterinary Clinical Complex, Lala Lajpat Rai University of Veterinary and Animal Sciences (LUVAS), Hisar between January, 2024 and January, 2025. A total of 55 dogs exhibiting clinical signs of respiratory illness were included in the study, comprising 38 males and 17 females. The majority of the dogs were between 1 and 3 years of age (47.27%). Coughing was the most commonly observed clinical sign (100%), followed by nasal discharge (60%), inappetence to anorexia (45.45%), and sneezing (40%).The majority of dogs were presented with incomplete vaccination status (43.63%). Clinical profile of dogs, sex- wise occurrence, age-wise occurrence and vaccination status of dogs in study is summarized in **Tables 2**–**5**. Dogs exhibiting respiratory signs such as coughing, nasal discharge or clinical evidence of bronchopneumonia were included in the study. Cases with respiratory symptoms attributable to cardiovascular disorders, tracheal dysfunction or allergic conditions were excluded. The extraction of DNA from swab samples were also done using the DNA extraction kit (HiPurA^®^ mammalian Genomic DNA Purification Kit, Kennett Square, PA, USA) according to manufacturer’s recommendation and TRIzol method was used for RNA extraction. Briefly, swab samples were dissolved in 400 μL of PBS for 15 minutes. The supernatant was centrifuged at 10,000 rpm for 10 minutes to remove any debris. For DNA extraction, 200 μL HiPurA^®^ Genomic lysis/binding buffer was added in 200 μL of sample supernatant along with 20 μL of Proteinase K. For RNA extraction, 200 μL of same sample supernatant was mixed with 300 μL of TRIzol reagent and vortexed vigorously. In this mixture, 120 μL of chloroform was added followed by vigorous vortexing to avoid formation of insoluble aggregates. Rest of the procedure for DNA and RNA extraction was same as mentioned in section 2.3. To assess the reliability of the developed multiplex PCR for clinical application, its performance was compared with a previously established PCR assay for CDV, for CAV-2 and for Bb (Hozbor et al., 1999; Agnihotri et al., 2017; Raja et al., 2021). The key diagnostic parameters including sensitivity, specificity, positive predictive value (PPV), and negative predictive value (NPV) were calculated. A chi-square test (with Yates’ continuity correction) was conducted using SPSS software (version 23.0) to assess the statistical significance of differences in pathogen detection rates between the two assays.

**Table 2:** Clinical profile of dogs with CIRDC.

Clinical sign	Cases depicting clinical sign (n=55)	% cases depicting clinical sign
Cough	55	100
*Non-Productive*	*44*	*80*
*Productive*	*11*	*20*
NasalDischarge	33	60
*Mucoid*	*22*	*40*
*Purulent*	*06*	*10.9*
*Mucopurulent*	*05*	*9*
Anorexia/Inappetence	25	45.45
Sneezing	22	40

## 3 Results

### 3.1 Optimization of simplex assay

Simplex PCR assays were optimized individually for CDV, CAV-2 and Bb by systematically adjusting annealing temperature, extension/annealing time, and primer concentration. For CDV, optimal conditions included an annealing temperature of 55°C, 30 s annealing/extension time, and 0.4 µM primer concentration, yielding a 274 bp amplicon. For CAV-2, the ideal parameters were 60°C annealing temperature, 30 s annealing/extension, and 0.4  µM primers, producing a 451 bp product. Similarly, the Bb assay was optimized at 60°C with 30 s annealing/extension time and 0.4  µM primers, amplifying a 672 bp fragment. All optimizations were confirmed via 1.5% agarose gel electrophoresis.

### 3.2 Optimization of multiplex PCR

Optimization of the assay resulted in the successful amplification of all target fragments: 274 bp for CDV, 451 bp for CAV-2 and 672 bp for Bb. Gradient PCR conducted across a temperature range of 45°C to 60°C demonstrated the most distinct and intense amplification at 55°C, which was subsequently selected as the optimal annealing temperature (**Supplementary Figure S1**). To refine incubation conditions, amplification was assessed at 55°C for 15, 30, and 45 seconds. Although all expected amplicons were detected at all time points, the strongest signal intensities were observed at 30 seconds, which was chosen as the optimal incubation time (**Supplementary Figure S2**). Primer concentration was further optimized through systematic evaluation. Initial multiplex reactions using various primer combinations identified 0.4 µM as the most effective concentration for each primer set. (**Supplementary Figures S3**-**S5**). Consistently, this concentration produced the most robust and specific amplification of all three targets, and was therefore selected as the final optimized primer concentration.

### 3.3 Analytical sensitivity, specificity and reproducibility

**Figure 1:**
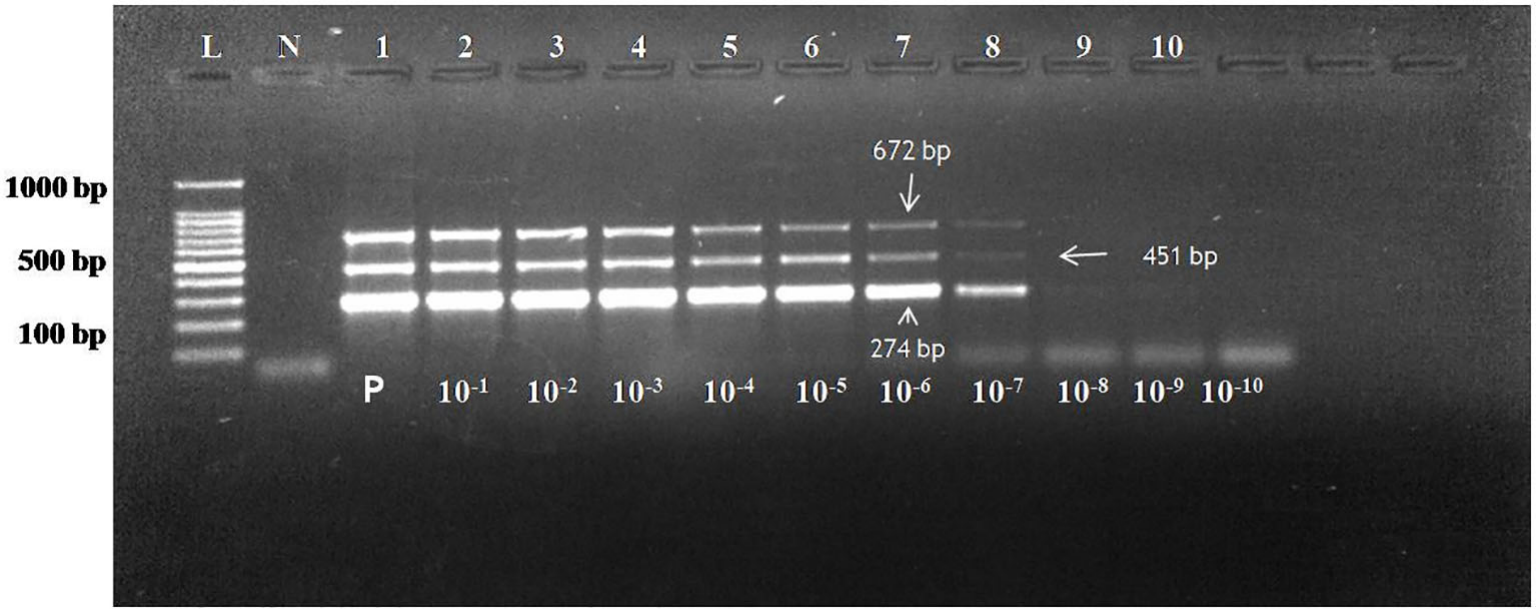
Agarose gel electrophoresis of PCR assay reaction with plasmid dilutions of CDV (274 bp), CAV-2 (451 bp) and Bb (672 bp) using 1.5% gel. Lane L: 100bp ladder; Lane N: Negative control; The concentration of plasmid in lane 1 is CDV plasmid 106x 10^9^copies/μL (CDV plasmid), 114.03 x 10^9^copies/μL (CAV 2 plasmid) and 110.16x 10^9^copies/μL(Bb plasmid) Lane 1 to 10: 10-fold serial dilution of the plasmid. Sensitivity of assay: 1060 copies/µl for CD (9^th^ dilution), 11403 copies/µl for CAV-2 (8^th^ dilution) and 11016 copies/µl for Bb till 8^th^ dilution.

For the detection of the sensitivity of the assay, copy number of all the three pathogens were calculated. The initial undiluted plasmid concentration of CDV, CAV-2 and Bb was 38.2 ng/μL, 61.4 ng/μL and 49.1 ng/μL. For multiplex assay, the copy number of all the three plasmids was calculated as per formula described in section 2.8. The copy number of CDV, CAV-2 and Bb in undiluted sample were 10.6 x 10^9^copies/μL, 16.29x 10^9^copies/μL and Bb were 12.24 x 10^9^copies/μL. Theses plasmids were further mixed in equivalent copy number to avoid the effect of size of different products. To make the copy number same the following concentrations were used 10 μL of CDV plasmid= 106x 10^9^copies/μL, 7 μL of CAV 2 plasmid= 114.03 x 10^9^copies/μL and 9 μL of Bb plasmid= 110.16x 10^9^copies/μL The sensitivity of the multiplex PCR was tested by detection of the three plasmids in serial dilutions expressed in copy number and was calculated as 1,060 copies/µL for CDV, 11,403 copies/µL for CAV-2 and 11,016 copies/µL for Bb based on standardized input plasmid concentrations (**Figure 1**). These sensitivity experiments were done in triplicate and were repeated with different dilutions prepared at different times as well as by different persons. The intra and inter-assay evaluations, both demonstrated consistent and comparable results across replicates at different intervals. Intra- and inter-assay evaluations, performed using three dilutions near the detection limit, exhibited a coefficient of variation (CV) below 5%, confirming the assay’s precision and robustness.

**Figure 2:**
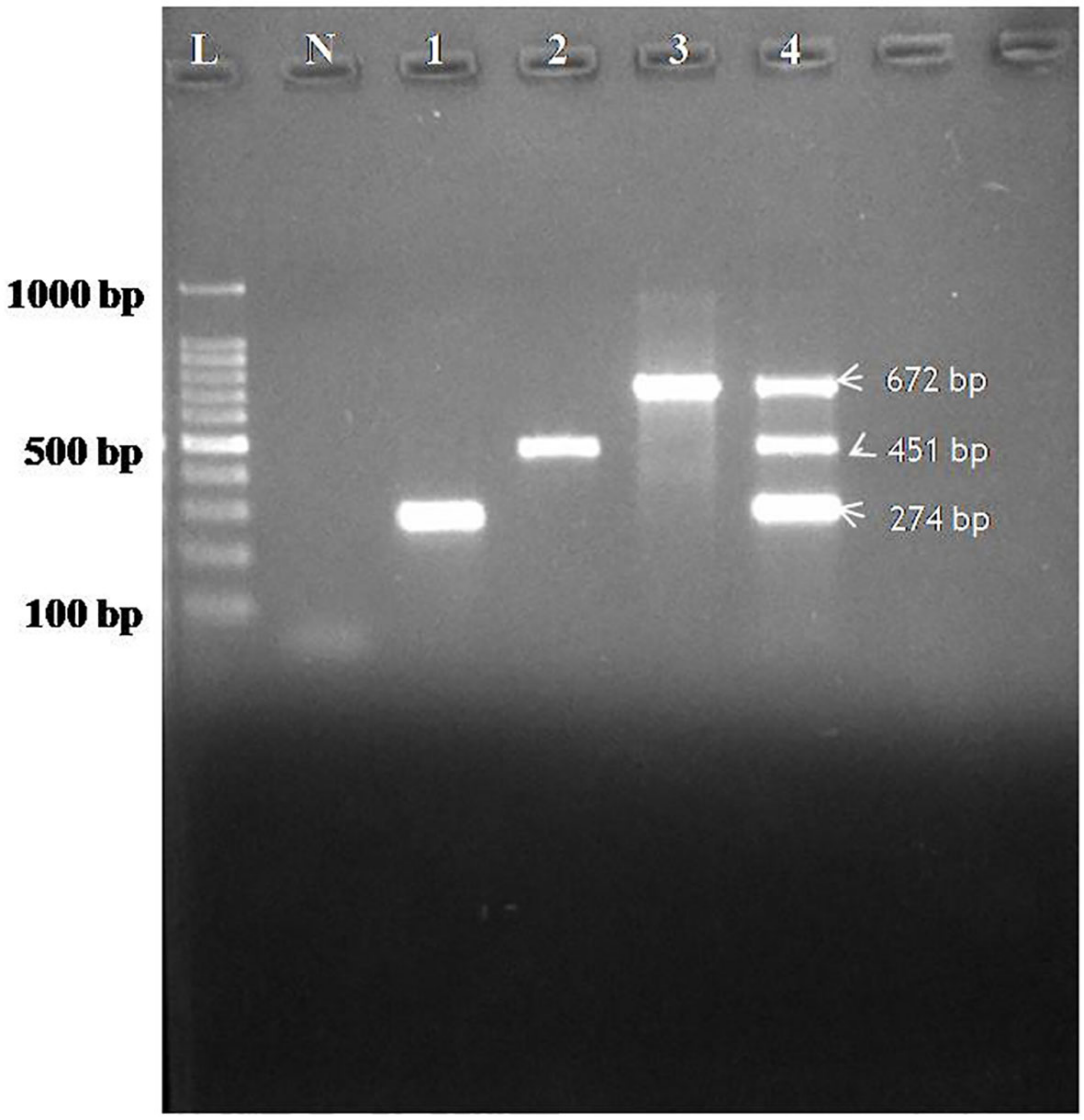
Agarose gel electrophoresis showing specificity of the assay performed with designed primers for CDV, CAV-2 and Bb. Lane L: 100 bp ladder; Lane N: Negative control; Lane 1: Specificity for CDV (274bp), Lane 2: Specificity for CAV-2 (451bp), Lane 3: Specificity for Bb(672), 4(M)- Multiplex specificity among plasmids showing all the three bands of CDV (274 bp), CAV-2 (451 bp) and Bb (672 bp).

Specificity of the assay was evaluated using individual plasmids for CDV, CAV-2 and Bb each yielding amplification exclusively at the expected product sizes with no cross-reactivity observed, thereby confirming 100% analytical specificity of the multiplex PCR assay (**Figure 2**).

### 3.4 Validation of multiplex PCR assay

**Figure 3:**
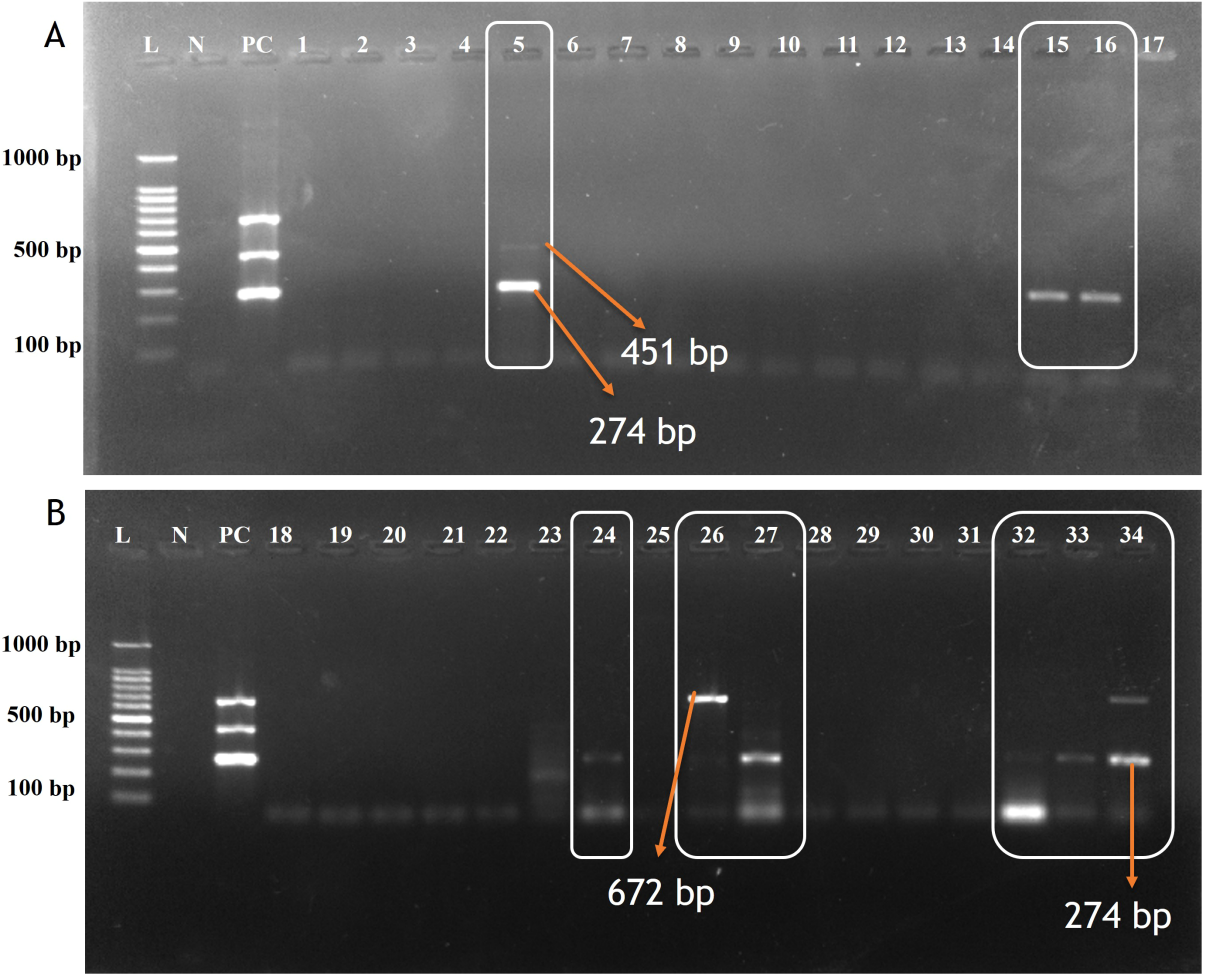
Agarose gel electrophoresis of screened samples using Multiplex PCR assay for detecting CDV, CAV-2 and Bb. **(A)** Lane L: 100 bp ladder; Lane N: Negative control; Lane PC: Positive control. Lane 5: Positive for CAV-2 (451bp) & CDV (274bp); Lane: 15,16: Positive for CDV(274bp); Lane 1,2,3,4,6,7,8,9,10,11,12,13,14,17 Negative samples. **(B)** Lane: 24, 27, 32, 33, 34: Positive for CDV(274bp); Lane 26 and 34: Positive for Bb (672 bp). Lane: 18,19,20,21,22,28,29,30 & 31: Negative samples.

A total of 55 oropharyngeal swab samples from dogs suspected of CIRDC were screened using the developed multiplex PCR assay for the simultaneous detection of CDV, CAV-2 and Bb. The samples were tested initially individually with standardized singlex PCR methods to confirm the reliability of assay (Hozbor et al., 1999; Agnihotri et al., 2017; Raja et al., 2021). For each swab sample 3 µL (10–50 ng/µL) of extracted DNA/cDNA was added to each multiplex PCR reaction. Out of the 55 samples tested, 18 (32.7%) tested positive using the developed multiplex PCR assay while 17 (30.9%) were found positive with singlex assay. CDV was the most frequently detected pathogen, identified in 14 samples (25%) as a single infection. Dual infections were observed in 2 samples (3.6%) with CDV and Bb, and in 1 sample (1.8%) with CDV and CAV-2. Additionally, Bb was detected alone in 1 sample (1.8%) (**Table 6**). The amplification of representative samples is shown in **Figure 3**. The previously established singlex PCR assay identified CDV DNA in 14 samples (25%) as a single infection, 2 samples (3.63%) with CAV-2 and 1 sample (1.8%) was detected for Bb. The 2x2 contingency table used for evaluation of diagnostic assay is given in **Supplementary Tables S2**-**S4**. The key diagnostic parameters are given in **Table 7**. Application of the chi-square test revealed a statistically significant association between the two assays, with chi-square values of 41.762 for CDV, 13.245 for CAV-2 and 37.318 for Bb (critical χ² = 3.84, df = 1). The high chi-square value indicating a highly significant association (p < 0.0001) between the two variables. These findings suggested that both assays exhibited comparable performance in detecting CIRDC pathogens.

**Table 6:** Results of application of multiplex PCR assay on 55 samples for detection of CIRDC pathogens.

Test	No of positive samples						No. of negative samples	Total no. of animals
	CDV only	CAV-2 only	Bb only	CDV + CAV-2	CAV-2 + Bb	CDV + Bb		
Multiplex PCR assay	14	–	01	01	–	02	37	55
Singlex PCR (Hozbor et al., 1999; Agnihotri et al., 2017; Raja et al., 2021)	12	–	01	01	–	02	39	55

**Table 7:** Evaluation of the multiplex PCR for different CIRDC pathogens.

Statistic	Value for CDV	95% CI for CDV	Value for CAV-2	95% CI for CAV-2	Value for Bb	95% CI for Bb	Overall evaluation of assay	95% CI
Sensitivity	100%	78.20% to 100%	100.00%	2.50% to 100.00%	100.00%	29.24% to 100.00%	94.12%	71.31% to 99.85%
Specificity	95%	83.08% to 99.39%	100.00%	93.40% to 100.00%	100.00%	93.15% to 100.00%	94.74%	82.25% to 99.36%
Positive Likelihood Ratio	20.00	5.18 to 77.21					17.88	4.62 to 69.27
Negative Likelihood Ratio	0.00	0.00	0.00	0.00	0.00	0.00	0.06	0.01 to 0.42
Disease prevalence (*)	27.27%	16.14% to 40.96%	1.82%	0.05% to 9.72%	5.45%	1.14% to 15.12%	30.91%	
Positive Predictive Value (*)	88.24%	66.02% to 96.66%	100.00%	2.50% to 100.00%	100.00%	29.24% to 100.00%	88.89%	67.38% to 96.87%
Negative Predictive Value (*)	100%	90.75% to 100%	100.00%	93.40% to 100.00%	100.00%	93.15% to 100.00%	97.30%	84.30% to 99.59%
Accuracy (*)	96.36	87.47% to 99.56%	100.00%	93.51% to 100.00%	100.00%	93.15% to 100.00%	94.55%	84.88% to 98.86%

(*) These values are dependent on disease prevalence.

## 4 Discussion

Canine Infectious Respiratory Disease Complex (CIRDC) remains a significant health concern, particularly in puppies and immunocompromised dogs, due to its multifactorial etiology, high transmission potential, and frequent involvement of viral co-infections. The present study aligns with previous research efforts aimed at optimizing molecular diagnostic platforms for three important CIRDC pathogens (Piewbang et al., 2016). While earlier studies emphasized optimizing annealing temperatures for separate panels of RNA and DNA viruses, this study fine tuned critical parameters such as annealing temperature, extension time, and primer concentration to achieve effective multiplexing for both DNA and RNA at same platform (Piewbang et al., 2016). Assay performance was maximized by targeting conserved gene regions and adjusting thermocycling parameters (Hao et al., 2019; Zhao et al., 2024). Primer optimization reduced cross-reactivity and improved multiplex assay efficiency (Maboni et al., 2019; Dong et al., 2022). In line with these findings, the present study successfully developed and standardized a multiplex PCR assay for CDV, CAV-2, and Bb. The optimal annealing temperature was determined to be 55 °C, with an extension time of 30 seconds. A primer concentration of 0.4 μM was standardized for all three targets. This assay offers a rapid and effective diagnostic tool for the simultaneous detection of key CIRDC pathogens, thereby supporting improved clinical decision-making and disease control.

Target gene selection was guided by previous studies highlighting diagnostic reliability and genetic conservation. The hemagglutinin (H) gene of CDV has demonstrated utility for both detection and genotyping due to its variability and lineage (Liu et al., 2015). Hemagglutinin (H) gene based diagnostic assay may provide important information on CDV evolution, possible vaccine failure, and virus jumping between host species (Iwatsuki et al., 2000; Becker et al., 2023). This gene is also the most commonly used gene for CDV phylogenetic categorization (Martella et al., 2008; Piewbang et al., 2016; Nguyen et al., 2017; Duque-Valencia et al., 2019). Despite being highly conserved, the nucleocapsid (N) gene does not contribute to a useful sequence analysis tool. At the molecular level, the N gene performs basic screening and detection and less effective for distinguishing CDV from other related viruses. In addition to evolutionary analysis, the H gene exhibits greater sequence variability than N gene, making it a suitable target for differentiating field strains and ensuring assay specificity. For CAV-2, the E3 region which differs significantly from that of CAV-1 enables precise strain differentiation and reflects viral evolution (Ramidi et al., 2020; Syamily et al., 2023). Detection of *Bordetella bronchiseptica* was enhanced by targeting the species-specific bfrZ gene, which is crucial for iron acquisition and highly conserved among isolates (Brickman and Armstrong, 2009; Jinnerot et al., 2015). The development of a multiplex PCR assay capable of simultaneously detecting three pathogens Canine Distemper Virus (CDV), Canine Adenovirus Type 2 (CAV-2), and *B. bronchiseptica* (Bb) represents a significant advancement in the molecular diagnosis of CIRDC.

Sensitivity and specificity are critical parameters in evaluating the diagnostic accuracy of molecular assays such as multiplex PCR, especially for detecting CIRDC pathogens. Several studies have demonstrated the high sensitivity of multiplex PCR assays for a wide range of CIRDC agents. One study reported an overall sensitivity of over 87%, including 100% sensitivity for CDV and CAV-2, although rapid tests failed to detect canine influenza virus (CIV) in 83 out of 102 PCR-positive samples (Piewbang et al., 2016). A 96.53% agreement between multiplex and singlex PCR was achieved, demonstrating high diagnostic sensitivity for *Mycoplasma canis* and *M. cynos* (Maboni et al., 2019). Nearly, 100% sensitivity was reported using nasal swabs, with performance found to be comparable to both RT-PCR and rapid antigen tests (Jeoung et al., 2013). High analytical sensitivity was observed in multiplex PCR, with detection limits of 10 copies/μL for CRCoV and CIV, and 100 copies/μL for CDV and CPiV (Syamily et al., 2023; Zhao et al., 2024). Additionally, the capability of PCR to detect co-infections in 43.2% of 139 positive cases out of 740 samples has been demonstrated (Jinnerot et al., 2015). These findings consistently underscore multiplex PCR’s ability to detect low pathogen loads and multiple agents in a single reaction. The sensitivity of developed mPCR was measured at 1,060 copies/μL for CDV, 11,403 copies/μL for CAV-2, and 11,016 copies/μL for Bb, indicating reliable detection of low copy numbers.

In terms of specificity, multiplex PCR has demonstrated 100% specificity, with no cross-reactivity observed across six target viruses (Piewbang et al., 2016). The assay also showed superior specificity with intra- and inter-assay variability below 5%, confirming robustness and reproducibility (Hao et al., 2019; Zhao et al., 2024). The presence of high concentrations of certain pathogens did not interfere with the detection of others, reinforcing specificity in complex samples (Dong et al., 2022). Consistent with these findings, the multiplex PCR assay developed in the present study targeting CDV, CAV-2, and Bb demonstrated 100% specificity. These results support the assay’s utility as a reliable, accurate, and efficient tool for both clinical diagnostics and epidemiological surveillance of CIRDC pathogens.

Sample type and timing also play critical roles in the diagnostic sensitivity of respiratory PCR assays. Both nasal and oropharyngeal swabs reflect the primary routes of viral shedding (Piewbang et al., 2016). A similar dual-site sampling approach was adopted in this study to optimize detection sensitivity, particularly for pathogens like CAV-2 that replicate preferentially in the lower respiratory tract. However, pathogen detection may still be influenced by the stage of disease and sample quality—limitations that are shared by all PCR-based diagnostics and should be considered in surveillance protocols. Out of 55 samples screened using the developed Multiplex PCR assay, 18 were found positive for the targeted pathogens, including both mono-infections and co-infections. Among the 18 positive samples, 14 (25%) were positive for CDV only, 2 (3.6%) were co-infected with CDV and Bb, 1 (1.8%) was co-infected with CDV and CAV-2, and 1 (1.8%) was positive for Bb only. Coughing was identified as the most prevalent clinical manifestation, being observed in all affected dogs, with non-productive coughing noted as the dominant type. Other commonly recorded signs included nasal discharge, reduced appetite (ranging from inappetence to anorexia), sneezing, ocular discharge, fever or pyrexia, and dyspnea. These clinical presentations were found to be consistent with previous findings (Ayodhya et al., 2013; Köse et al., 2021). The observed cough was likely caused by airway irritation resulting from inflammation or infiltration of the lower respiratory tract. The presence of nasal discharge was attributed to inflammatory changes within the nasal mucosa and nares. Furthermore, signs of exercise intolerance observed in some cases were considered to be associated with compromised respiratory function and reduced ventilatory efficiency.

While this assay represents a significant advancement, some limitations remain. First, the inability to differentiate vaccine strains from field strains—particularly for modified live vaccines (MLVs)—may result in false positives in recently vaccinated animals (Ruch-Gallie et al., 2016). Second, although the assay targets three major CIRDC agents, pathogens such as CPIV, CRCoV, CaHV-1, *Mycoplasma cynos*, and *Streptococcus equi subsp. zooepidemicus* also play important roles in CIRDC pathogenesis and should be considered in future assay iterations. Expanding the current platform to include these additional agents while maintaining high analytical performance is both feasible and necessary.

## 5 Conclusion

In conclusion, the multiplex PCR assay described herein offers a rapid, robust, and clinically valuable tool for the simultaneous detection of CDV, CAV-2, and Bb. By focusing on the most prevalent and impactful CIRDC pathogens, the assay addresses a critical need for efficient, high-throughput diagnostics in both routine and outbreak scenarios. It compares favorably to, and builds upon, existing multiplex systems, offering enhanced specificity, sensitivity, and operational simplicity. Taken together, our findings contribute meaningfully to the evolving landscape of canine respiratory diagnostics and reinforce the growing role of multiplex PCR as a frontline diagnostic modality in veterinary medicine.

## Data Availability

The original contributions presented in the study are included in the article/**Supplementary Material**. Further inquiries can be directed to the corresponding authors.

## References

[B1] Agnihotri D. Singh Y. Maan S. Jain V. K. Kumar A. Sindhu N. . (2017). Molecular detection and clinico-haematological study of viral gastroenteritis in dogs. Haryana Vet. 56, 72–76.

[B2] Ayodhya S. Rao D. T. Reddy Y. N. Sundar N. S. Kumar V. G. (2013). Epidemiological, clinical and haematological studies on canine respiratory diseases in and around Hyderabad city, Andhra Pradesh, India. Int. J. Curr. Microbiol. Appl. Sci. 2, 453–462.

[B3] Becker A. S. Silva Júnior J. V. J. Weiblen R. Flores E. F. (2023). An appraisal of gene targets for phylogenetic classification of canine distemper virus: Is the hemagglutinin the best candidate? Virus Res. 325, 199043. doi: 10.1016/j.virusres.2023.199043, PMID: 36634899 PMC10194282

[B4] Brickman T. J. Armstrong S. K. (2009). Temporal signaling and differential expression of Bordetella iron transport systems: the role of ferrimones and positive regulators. Biometals 22, 33–41. doi: 10.1007/s10534-008-9189-9, PMID: 19130264 PMC3207241

[B5] Buonavoglia C. Martella V. (2007). Canine respiratory viruses. Vet. Res. 38, 355–373. doi: 10.1051/vetres:2006058, PMID: 17296161

[B6] Day M. J. Carey S. Clercx C. Kohn B. Marsilio F. Thiry E. . (2020). Aetiology of canine infectious respiratory disease complex and prevalence of its pathogens in Europe. J. Comp. Pathol. 176, 86–108. doi: 10.1016/j.jcpa.2020.02.005, PMID: 32359641 PMC7103302

[B7] Dong J. Tsui W. N. T. Leng X. Fu J. Lohman M. Anderson J. . (2022). Development of a three-panel multiplex real-time PCR assay for simultaneous detection of nine canine respiratory pathogens. J. Microbiol. Methods 199, 106528. doi: 10.1016/j.mimet.2022.106528, PMID: 35753509

[B8] Duque-Valencia J. Sarute N. Olarte-Castillo X. A. Ruíz-Sáenz J. (2019). Evolution and interspecies transmission of canine distemper virus-an outlook of the diverse evolutionary landscapes of a multi-host virus. Viruses 11, 582. doi: 10.3390/v11070582, PMID: 31247987 PMC6669529

[B9] Ellis J. A. (2015). How well do vaccines for Bordetella bronchiseptica work in dogs? A critical review of the literature 1977–2014. Vet. J. 204, 5–16. doi: 10.1016/j.tvjl.2015.02.006, PMID: 25747699

[B10] Erles K. Dubovi E. J. Brooks H. W. Brownlie J. (2004). Longitudinal study of viruses associated with canine infectious respiratory disease. J. Clin. Microbiol. 42, 4524–4529. doi: 10.1128/JCM.42.10.4524-4529.2004, PMID: 15472304 PMC522361

[B11] Hao X. Liu R. He Y. Xiao X. Xiao W. Zheng Q. . (2019). Multiplex PCR methods for detection of several viruses associated with canine respiratory and enteric diseases. PloS One 14, e0213295. doi: 10.1371/journal.pone.0213295, PMID: 30830947 PMC6398926

[B12] Hozbor D. Fouque F. Guiso N. (1999). Detection of Bordetella bronchiseptica by the polymerase chain reaction. Res. Microbiol. 150, 333–341. doi: 10.1016/S0923-2508(99)80059-X, PMID: 10422694

[B13] Islahi S. Sen M. Das A. Gupta A. Trivedi S. Agarwal J. (2019). Bordetella bronchiseptica infection in an intensive care unit patient. MGM J. Med. Sci. 6, 152–154. doi: 10.4103/mgmj.mgmj_13_20

[B14] Iwatsuki K. Tokiyoshi S. Hirayama N. Nakamura K. Ohashi K. Wakasa C. . (2000). Antigenic differences in the H proteins of canine distemper viruses. Vet. Microbiol. 71, 281–286. doi: 10.1016/s0378-1135(99)00172-8, PMID: 10703710

[B15] Jeoung H. Y. Song D. S. Jeong W. S. Lee W. H. Song J. Y. An D. J. (2013). Simultaneous detection of canine respiratory disease-associated viruses by a multiplex reverse transcription polymerase chain reaction assay. J. Vet. Med. Sci. 75, 103–106. doi: 10.1292/jvms.12-0287, PMID: 22971595

[B16] Jinnerot T. Malm K. Eriksson E. Wensman J. J. (2015). Development of a TaqMan real-time PCR assay for detection of Bordetella bronchiseptica. Vet. Sci. Res. Rev. 1, 14–20.

[B17] Keil D. J. Fenwick B. (1998). Role of Bordetella bronchiseptica in infectious tracheobronchitis in dogs. J. Am. Vet. Med. Assoc. 212, 200–207. doi: 10.2460/javma.1998.212.02.200, PMID: 9448823

[B18] Köse Sİ Maden M. Sayin Z. (2021). Clinical and bacteriological analysis of respiratory tract infections in sheltered dogs and determination of antibacterial treatment options. J. Hellenic Vet. Med. Soc 72, 3491–3502. doi: 10.12681/jhvms.29441

[B19] Liu D. F. Liu C. G. Tian J. Jiang Y. T. Zhang X. Z. Chai H. L. . (2015). Establishment of reverse transcription loop-mediated isothermal amplification for rapid detection and differentiation of canine distemper virus infected and vaccinated animals. Infect. Genet. Evol. 32, 102–106. doi: 10.1016/j.meegid.2015.03.002, PMID: 25769803 PMC7106007

[B20] Maboni G. Seguel M. Lorton A. Berghaus R. Sanchez S. (2019). Canine infectious respiratory disease: new insights into the etiology and epidemiology of associated pathogens. PloS One 14, e0215817. doi: 10.1371/journal.pone.0215817, PMID: 31022218 PMC6483346

[B21] Martella V. Elia G. Buonavoglia C. (2008). Canine distemper virus. Vet. Clin. North Am. Small Anim. Pract. 38, 787–797. doi: 10.1016/j.cvsm.2008.02.007, PMID: 18501278

[B22] Mattoo S. Cherry J. D. (2005). Molecular pathogenesis, epidemiology and clinical manifestations of respiratory infections due to Bordetella pertussis and other Bordetella subspecies. Clin. Microbiol. Rev. 18, 326–382. doi: 10.1128/CMR.18.2.326-382.2005, PMID: 15831828 PMC1082800

[B23] Nguyen D. V. Suzuki J. Minami S. Yonemitsu K. Nagata N. Kuwata R. . (2017). Isolation and phylogenetic analysis of canine distemper virus among domestic dogs in Vietnam. J. Vet. Med. Sci. 79, 123–127. doi: 10.1292/jvms.16-0394, PMID: 27746406 PMC5289248

[B24] Payungporn S. Chutinimitkul S. Chaisingh A. Damrongwatanapokin S. Buranathai C. Amonsin A. . (2006). Single-step multiplex real-time RT-PCR for H5N1 influenza A virus detection. J. Virol. Methods 131, 143–147. doi: 10.1016/j.jviromet.2005.08.004, PMID: 16183140

[B25] Pecoraro H. L. Spindel M. E. Bennett S. Lunn K. F. Landolt G. A. (2013). Evaluation of virus isolation, one-step real-time reverse transcription polymerase chain reaction assay, and two rapid influenza diagnostic tests for detecting canine influenza A virus H3N8 shedding in dogs. J. Vet. Diagn. Invest. 25, 402–406. doi: 10.1177/1040638713480500, PMID: 23536615

[B26] Piewbang C. Rungsipipat A. Poovorawan Y. Techangamsuwan S. (2016). Development and application of multiplex PCR assays for detection of virus-induced respiratory disease complex in dogs. J. Vet. Med. Sci. 78, 1847–1854. doi: 10.1292/jvms.16-0342, PMID: 27628592 PMC5240764

[B27] Posuwan N. Payungporn S. Thontiravong A. Kitikoon P. Amonsin A. Poovorawan Y. (2010). Prevalence of respiratory viruses isolated from dogs in Thailand during 2008–2009. Asian Biomed. 4, 563–569. doi: 10.2478/abm-2010-0071

[B28] Radtanakatikanon A. Keawcharoen J. Charoenvisal N. T. Poovorawan Y. Prompetchara E. Yamaguchi R. . (2013). Genotypic lineages and restriction fragment length polymorphism of canine distemper virus isolates in Thailand. Vet. Microbiol. 166, 76–83. doi: 10.1016/j.vetmic.2013.05.015, PMID: 23830775

[B29] Raja P. Sachin V. Parthiban M. Janaki P. A. (2021). Molecular characterization of canine adenovirus type 2 in dogs from India. Virus Dis. 32, 369–374. doi: 10.1007/s13337-021-00690-7, PMID: 33969151 PMC8096630

[B30] Ramidi A. Ganji V. K. Buddala B. Yella N. R. Manthani G. P. Putty K. (2020). E3 gene-based genetic characterization of canine adenovirus-2 isolated from cases of canine gastroenteritis in India revealed a novel group of the virus. Intervirology 62, 216–221. doi: 10.1159/000507329, PMID: 32259812

[B31] Reagan K. L. Sykes J. E. (2020). Canine infectious respiratory disease. Vet. Clin. Small Anim. Pract. 50, 405–418. doi: 10.1016/j.cvsm.2019.10.009, PMID: 31813556 PMC7132485

[B32] Ruch-Gallie R. Moroff S. Lappin M. R. (2016). Adenovirus 2, Bordetella bronchiseptica, and parainfluenza molecular diagnostic assay results in puppies after vaccination with modified live vaccines. J. Vet. Intern. Med. 30, 164–166. doi: 10.1111/jvim.13821, PMID: 26692461 PMC4913651

[B33] Sariga K. Bipin K. C. Deepa P. M. Rateesh R. L. Dinesh P. T. (2022). Molecular identification and occurrence of bordetellosis among dogs in northern Kerala. J. Vet. Res. 12, 123–135. doi: 10.51966/jvas.2022.53.4.541-544

[B34] Suwannakarn K. Payungporn S. Chieochansin T. Samransamruajkit R. Amonsin A. Songserm T. . (2008). Typing (A/B) and subtyping (H1/H3/H5) of influenza A viruses by multiplex real-time. J. Virol. Methods 152, 25–31. doi: 10.1016/j.jviromet.2008.06.002, PMID: 18598722

[B35] Syamily S. Rajasekhar R. Anoopraj R. Ravishankar C. Jishnu H. P. (2023). Detection and molecular characterisation of canine adenovirus type 1 from a fatal case of infectious canine hepatitis from India. Vet. Rec Case Rep. 11, e698. doi: 10.1002/vrc2.698

[B36] Wille M. Wensman J. J. Larsson S. van Damme R. Theelke A. K. Hayer J. . (2020). Evolutionary genetics of canine respiratory coronavirus and recent introduction into Swedish dogs. Infect. Genet. Evol. 82, 104290. doi: 10.1016/j.meegid.2020.104290, PMID: 32205264 PMC7102562

[B37] Yondo A. Kalantari A. A. Fernandez-Marrero I. McKinney A. Naikare H. K. Velayudhan B. T. (2023). Predominance of canine parainfluenza virus and Mycoplasma in canine infectious respiratory disease complex in dogs. Pathogens 12, 1356. doi: 10.3390/pathogens12111356, PMID: 38003820 PMC10675171

[B38] Zhao D. Sun Y. Guo J. Tang Y. Wang Z. Wen X. . (2024). Pathogenic characteristics of an infection with canine influenza virus and Streptococcus equi subsp. zooepidemicus alone or in combination in mice. Transbound Emerg. Dis. 2024, 2237621. doi: 10.1111/tbed.15088 40303164 PMC12016976

