# Supplementary material for: Development of Multiplex PCR assay for detection of Canine Infectious Respiratory Disease Complex (CIRDC) pathogens in dogs
Source: Front Cell Infect Microbiol. 2025 Nov 28;15:1661499. doi: 10.3389/fcimb.2025.1661499 (PMC12698605; doi:10.3389/fcimb.2025.1661499)
Supplement: Supplementary file 1 [file Supplementaryfile1.docx]

**
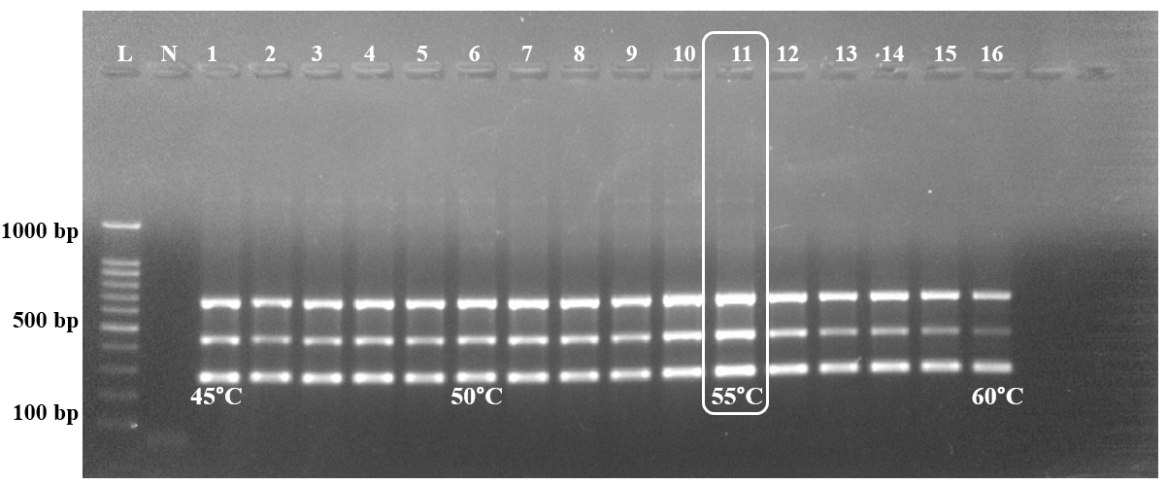
**

**Fig. 1: Agarose gel electrophoresis of Multiplex PCR amplified products of CDV (274 bp), CAV-2 (451 bp) and Bb (672 bp)** **for optimization of temperature using 1.5% gel. Lane L: 100bp ladder; Lane N: Negative control; Lane 1: 45°C; 2: 46°C; 3: 47°C; 4: 48°C; 5: 49°C; 6: 50°C; 7: 51°C; 8: 52°C; 9: 53°C; 10: 54°C; 11: 55°C; 12: 56°C; 13: 57°C; 14: 58°C; 15: 59°C; 16: 60°C.**

**Final optimized temperature for Multiplex PCR assay was 55°C.**

**
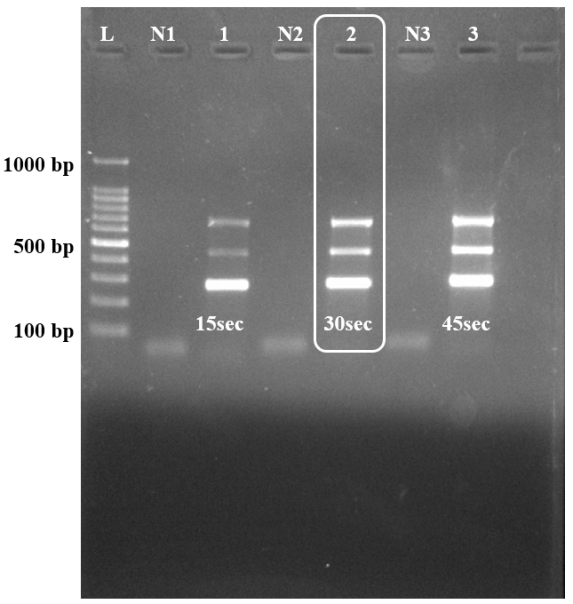
**

**Fig. 2: Agarose gel electrophoresis of Multiplex PCR reaction of CDV (274 bp), CAV-2 (451 bp) and Bb (672 bp)**  **for optimization of time using 1.5% gel. Lane L: 100bp ladder; Lane N1: Negative control 15sec; Lane 1: 15sec; Lane N2: Negative control 30sec; 2: 30sec; Lane N3: Negative control 45sec; Lane 3: 45sec.**

**Final optimized time for Multiplex PCR assay was 30sec.**


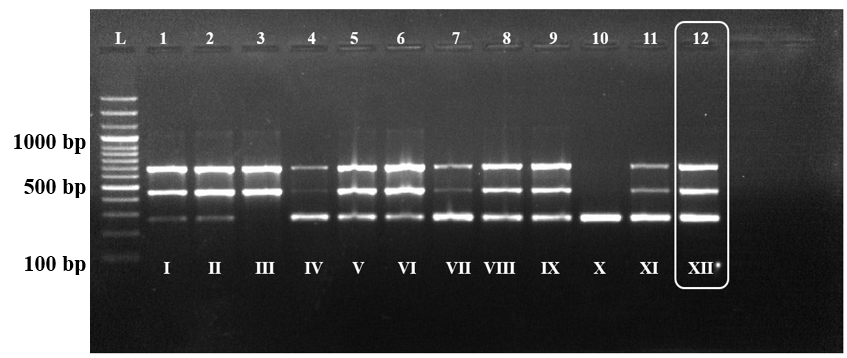


**Fig. 3: Agarose gel electrophoresis of Multiplex PCR reactionfor optimization of primer concentration of CDV (274 bp) using 1.5% gel. Lane L: 1 Kb ladder; Lane N: Negative control; Lane 1 to 12: I to XII primer concentrations (0.1-0.4 μM). Final optimized primer concentration for CDV in Multiplex PCR assay was 0.4 μM (XII).**

**
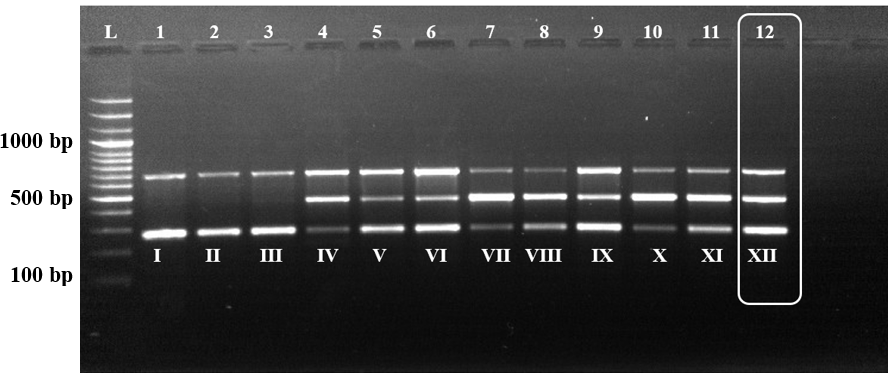
**

**Fig. 4:Agarose gel electrophoresis of Multiplex PCR reaction for optimization of primer concentration of CAV-2 (451 bp)** **using 1.5% gel. Lane L: 1 Kb ladder; Lane N: Negative control; Lane 1 to 12: I to XII primer concentrations (0.1-0.4 μM). Final optimized primer concentration for CAV-2 in Multiplex PCR assay was 0.4 μM (XII)**

**
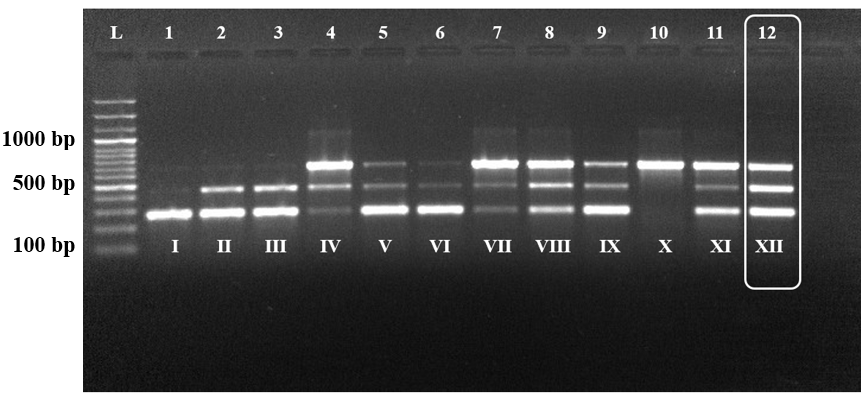
**

**Fig. 5:Agarose gel electrophoresis of Multiplex PCR reaction for optimization of primer concentration of Bb (672 bp)**  **using 1.5% gel. Lane L: 1 Kb ladder; Lane N: Negative control; Lane 1 to 12: I to XII primer concentrations (0.1-0.4 μM). Final optimized primer concentration for Bb in Multiplex PCR assay was 0.4 μM (XII)**
