# Supplementary material for: Development of Multiplex PCR assay for detection of Canine Infectious Respiratory Disease Complex (CIRDC) pathogens in dogs
Source: Front Cell Infect Microbiol. 2025 Nov 28;15:1661499. doi: 10.3389/fcimb.2025.1661499 (PMC12698605; doi:10.3389/fcimb.2025.1661499)
Supplement: Supplementary file 2 [file Table1.docx]

Supplementary tables for Manuscript entitle “**Development of Multiplex PCR assay for detection of Canine Infectious Respiratory Disease (CIRDC) pathogens in dogs”**

Table 1: List of sequences used for designing of primers of all the three CIRDC pathogens

| Species | Primer name | Accession numbers |
| --- | --- | --- |
| *Bordetella bronchiseptica* | BBV/BfrZ/F/1519-1538 and BBV/BfrZ/R/2170-2190 | AJ251793.1:1, AP014582.1, BX640451.1, CP020651.1, CP020819.1, CP014013.2, CP020650.1, CP019934.1, CP024173.1, CP024171.1, CP020649.1, CP020645.1, CP018761.1, CP050967.1, CP024175.1, CP020818.1, CP132332, HE965807.1, LR134480.1, |
| Canine Adenovirus | CAV2/E3/F/38-58 and CAV2/E3/R/464-488 | AC_000020.1, GQ915311.1, JX416841.1, JX416842.1, KF676978.1, KU315333.1, KU725671.1, KU725672.1, KU725673.1, KU725674.1, LC557011.1, MF356371.1, MN652565.1, MN652566.1, MT193154.1, MT193156.1, MT193157.1, MT892837.1, MW811330.1, OP018862.1, OP018872.1, OP060355.1, OP618116.1, OP644981.1, OQ596341.1, OR892272.1, PP027961.1, PP027962.1, S38212.1, SV431, SV435, SV521, SV675, U77082.1 |
| Canine distemper virus | CD/H/F/346-368 and CD/H/R/597-619 | AF112189.1, AF164967.1, AY964114.1, EF095750.1, FJ461702.1, FJ705238.1, GU266280.1, GU810819.1, HM563058.1, HM563059.1, JN008894.1, JN008902.1, JN153023.1, JN215475.1, JN215476.1, JN812975.1, JQ732170.1, KC479138.1, KC479139.1, KC479140.1, KC479141.1, KU578256.1, LC011103.1, MF964181.1, MF964190.1, MN335912.1, MT136724.1, MT292106.1, MT448054.1, MT932493.1, MW876862.1, OQ282897.1, OQ282900.1, OQ282901.1, OQ282902.1, OR123691.1, OR123692.1, OR243301.1, OR880598.1, OR880599.1, OR880600.1, OR880601.1 |

Table 2: Contingency table of CDV used for calculation of key diagnostic metrics.

|  | Simplex PCR assay | |  |
| --- | --- | --- | --- |
| Multiplex assay | Positive samples | Negative samples | Total |
| Positive samples | 15 | 2 | 17 |
| Negative samples | 0 | 38 | 38 |
| Total | 15 | 40 | 55(*n*) |

Table 3: Contingency table of CAV-2 used for calculation of key diagnostic metrics.

|  | Simplex PCR assay | |  |
| --- | --- | --- | --- |
| Multiplex assay | Positive samples | Negative samples | Total |
| Positive samples | 01 | 0 | 01 |
| Negative samples | 0 | 54 | 54 |
| Total | 01 | 54 | 55(*n*) |

Table4: Contingency table of *B. Bronchiseptica* used for calculation of key diagnostic metrics.

|  | Simplex PCR assay | |  |
| --- | --- | --- | --- |
| Multiplex assay | Positive samples | Negative samples | Total |
| Positive samples | 03 | 0 | 03 |
| Negative samples | 0 | 52 | 52 |
| Total | 03 | 52 | 55(*n*) |
